# Proximal Tibiofibular Joint Arthrodesis Due to Recurrent Giant Ganglion Cyst Causing Peroneal Nerve Palsy

**DOI:** 10.7759/cureus.34399

**Published:** 2023-01-30

**Authors:** Murat Birinci, Oguzhan Korkmaz, Bilal Bostanci, Tugrul Ormeci, Adnan Kara

**Affiliations:** 1 Department of Orthopaedics and Traumatology, Istanbul Medipol University, Istanbul, TUR; 2 Department of Radiology, Istanbul Medipol University, Istanbul, TUR; 3 Department of Orthopaedics and Traumatology, Istanbul Medipol University, İstanbul, TUR

**Keywords:** recurrence, peroneal nerve palsy, arthrodesis, drop foot, ganglion cyst

## Abstract

Ganglion cysts are masses that we encounter frequently in our daily practice, usually in the upper extremity, less frequently in the lower extremities, and rarely cause compression symptoms. We present a case of a massive ganglion cyst of the lower limb causing peroneal nerve compression, managed with excision and proximal tibiofibular joint arthrodesis to prevent recurrence.

Examination and radiological imaging of a 45-year-old female patient who was admitted to our clinic showed new-onset weakness in right foot movements and numbness on the dorsum of the foot and lateral cruris, a mass consistent with a ganglion cyst expanding the muscle was detected in the peroneus longus muscle. In the first surgery, the cyst was carefully resected. After three months, the patient came with a repeated mass on the lateral side of the knee. After confirmation of the ganglion cyst with clinical examination and MRI, a second surgery was planned for the patient. In this stage, we performed a proximal tibiofibular arthrodesis for the patient. Her symptoms recovered during the early follow-up period and no recurrence occurred during the two years of the follow-up period.

Although the treatment of ganglion cysts seems easy, it can sometimes be challenging. We think that arthrodesis may be a good treatment option in recurrent cases.

## Introduction

Synovial cysts may arise from any synovial joint, synovial-lined tendon sheath, or bursa. It is more common in the upper extremity and rarely causes peripheral nerve compression [1,2]. Around the knee joint, they are seen usually in the popliteal fossa of the tibiofemoral joint and may cause a wide range of compressive symptoms depending on the localization. 

Although drop foot secondary to peroneal nerve damage is mostly seen after acute injuries such as trauma, it may also develop on a chronic basis. Peroneal nerve palsy (PNP) may result from tumors (sarcoma), hematoma, and cysts [3,4]. As far as we know, in 1921, Sultan reported the first case of compression of the peroneal nerve by a synovial cyst [5]. In the current article, we present a huge synovial cyst compressing the common peroneal nerve and leading drop foot and numbness in the lower limb.

## Case presentation

A 45-year-old female was admitted to the outpatient clinic with progressive pain followed by swelling on the proximal lateral side of her right leg, which progressively increased during the past one month. For 10 years, the patient was complaining an episodic pain around this area. Additionally, for the past 15 days, she had numbness on the lateral side of her leg and drop foot developed in this duration. There was no history of trauma. Previously, a cyst was detected at the lateral side of the proximal tibia in an ultrasound (USG) examination performed in another center. Since she was suffering from intermittent moderate pain, analgesics were used regularly by the patient during this period. She applied to our hospital due to the numbness and drop foot developing in recent days.

On physical examination, swelling was seen along the peroneal muscles starting from the right fibular head up to the middle one-third of the tibia, and tenderness was palpated around the fibular head. There were sensory disturbances along the territory of the superficial peroneal nerve and palsy of muscles, dependent on the deep peroneal nerve. She was unable to evert or dorsiflex her foot and peripheral pulses were palpable.

**Figure 1:**
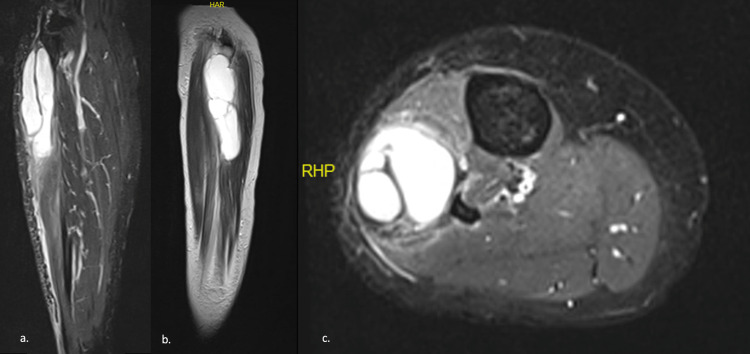
(a) Coronal, (b) sagittal, and (c) axial T2-weighted MRI images of the ganglion cyst

USG, MRI, and electromyogram (EMG) were performed on the patient. In USG examination, an anechoic cystic lesion was observed in the posterolateral area of the right cruris. Further investigation with MRI revealed a lobulated, multilocular, well-demarcated cystic lesion in the peroneus longus muscle, diffuse edema, and inflammation around this region. Its size was approximately 36*35*120 mm (Figure 1). EMG showed that there was no motor response in the extensor digitorum brevis and tibialis anterior muscles with peroneal nerve stimulation. To rule out other possible diagnoses, a Tru-Cut biopsy was performed. As no malignancy was detected in the specimens, the lesion was considered for surgical excision.

**Figure 2:**
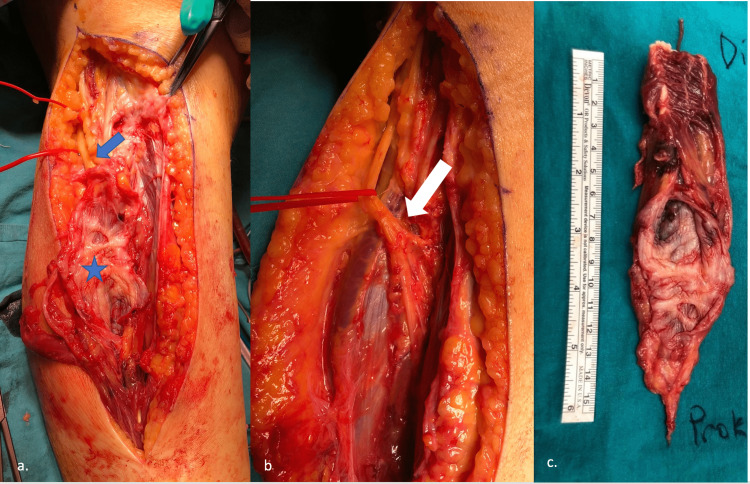
(a) Blue arrow shows the proximal part of peroneal nerve compressed by the peroneal muscles, blue star shows the intramuscular septation; (b) White arrow points intact peroneal nerve after removal of disrupted muscle; (c) Disrupted peroneus longus muscle

A 20 cm incision was made through the posterolateral surface of the cruris along the course of the fibular nerve. The nerve was found intact, 1.5 cm distal to the head of the fibula, but crushed due to the expansion in the peroneus longus muscle in the region where the common peroneal nerve divided into deep and superficial branches. Ganglion cyst content was seen at the proximal tibiofibular joint level. In accordance with MRI, it was observed that the peroneus longus muscle was enlarged and there were septa that were felt in the longitudinal plane for about 10 cm. Residual muscle structure was atrophic and afunctional. The mass containing the surface of the peroneus longus muscle was excised from a total of 15 cm, while preserving the peroneal nerve and its branches (Figure 2). The distal tendinous part was transferred to the peroneus brevis. The proximal tibiofibular joint surface was curetted. Histopathological evaluation showed “dense collagenous walls with foci of myxoid changes”, which confirmed the diagnosis of the cyst.

Postoperatively for the first three weeks, a short leg splint was applied, and for the following six weeks, a dynamic ankle-foot orthosis was applied. After one month of the treatment, transcutaneous electrical nerve stimulation (TENS) therapy was started by the physiotherapist. In the sixth week, ankle dorsiflexion and toe extension recovered. But the patient was not able to dorsiflex her first toe.

**Figure 3:**
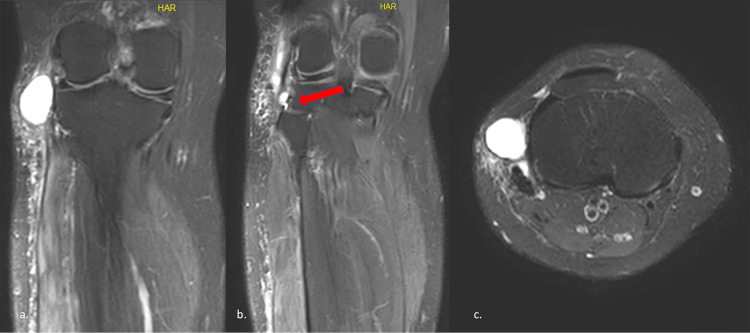
(a) and (b) Preoperative coronal MRI section showing cyst recurrence. Red arrows indicates the PTFJ connection; (c) Axial MRI section of ganglion cyst recurrence PTFJ: proximal tibiofibular joint

At the third month, a 3*3 cm, well-demarcated, mobile mass was palpable on the lateral side of the knee. No worsening was noticed in motor and sensory function. In the imaging performed with suspicion of recurrence, an appearance compatible with a ganglion cyst originating from the proximal tibiofibular joint (PTFJ) was observed. PTFJ arthrodesis was planned when the MRI revealed the ganglion cyst originating from the PTFJ (Figure 3).

**Figure 4:**
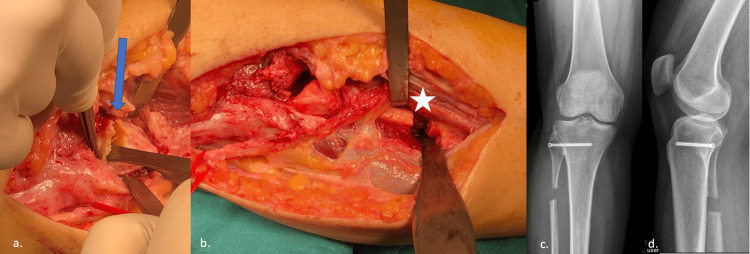
(a) Preparation of proximal tibiofibular joint. Resection of articular surface; (b) Fibular osteotomy; (c) and (d) Weightbearing anterioposterior and lateral plain radiographs on the second-year follow-up

A 12 cm incision was made over the old incision line under the tourniquet. After the subcutaneous tissue and adhesions were passed, the mass, whose contents were compatible with the synovial cyst, was released from the surrounding tissues up to the proximal tibiofibular joint. Fibular and tibial surfaces of the PTFJ were prepared for arthrodesis. Cartilage tissue was removed up to the subchondral bone. Then, approximately 1 cm of the fibular segment was resected from approximately 8 cm distal of the fibula head to allow joint movement. Tibiofibular arthrodesis was achieved with a 3.5 mm cannulated screw (Figure 4).

**Figure 5:**
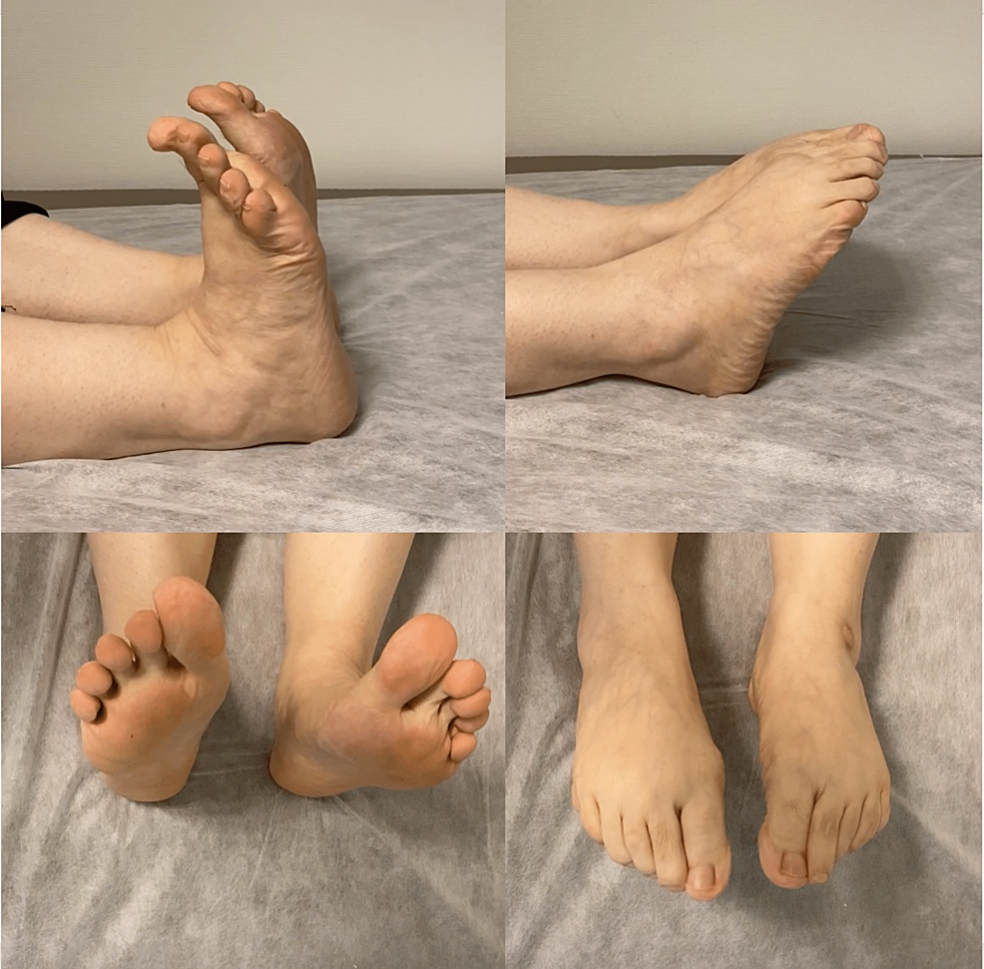
Second-year follow-up. Full range of motion on the physical examination

Partial weight bearing was allowed with a short leg splint and then for six weeks, dynamic ankle foot orthoses (AFO) again. After eight months from the first operation, the patient could fully dorsiflex and evert her ankle, and extend her toes. EMG revealed significant improvement in motor strength. On her second-year control examination, no recurrence, and no motor and sensorial loss was noted (Figure 5).

## Discussion

Synovial cysts are common soft tissue tumors. They usually appear in the upper extremity and may lead to ulnar and median nerve compression [6]. Although less common, there are cases in the literature that have been reported to cause nerve compression in the lower limbs. PNP may occur after variable reasons, such as trauma, tumor, cast complication, hematoma, or iatrogenic causes. Compression by synovial cysts is a rare reason for PNP [7,8]. In our case, PNP compression was detected due to compression of the synovial cyst within the peroneus longus muscle.

Synovial cysts appear in areas that are constantly exposed to mechanical stress. Most often they arise from periarticular soft tissue and also synovium and contain a clear, colorless, gelatinous fluid as it seems in our case. A synovial cyst may be intraneural and extra-neural. Most of the cysts that cause peroneal nerve compression are intraneural [9]. Extra-neural cysts involving peroneal muscle and compressing the peroneal nerve, similar to our case, have been described earlier [2,10].

Some imaging methods and EMG is required in establishing the diagnosis. EMG can be useful in determining the level of lesions and the severity of motor. Ganglia appear on MRI as lobulated, rounded, well-limited fluid collections. However, it is not always easy on MRI to distinguish a ganglion cyst from a giant cell tumor of synovial sheath or sarcoma [4,11]. USG can be useful to show the cystic structure of the lesion and differentiate them from solid tumors. A plain radiograph may be used to rule out bony anomalies and fractures.

To avoid misdiagnosis, it needs to be kept in mind that patients visiting the clinic with pain radiating along the anterolateral surface of the leg, hypoesthesia, and motor deficiency may have other reasons such as nerve compression at the lumbar level. Nikolopoulos et al. presented a patient who was initially evaluated as having spinal stenosis and was finally diagnosed with ganglion cyst at the lateral side of the proximal fibula as a result of further examinations after his complaints were not overcome with medical treatment [2]. Preoperative lumbar MRI was not correlated with nerve compression.

During excision, complications such as vascular and nerve injuries may occur during surgical dissection. Another complication is recurrence. Recurrence after cyst excision from the lower extremity has been reported in the literature as occurring in 13% of cases [12]. Also, in their series of nine cases, Lateur et al. stated that arthrodesis and 1 cm fibula excision gave superior results instead of recurrent cyst excision after recurrence [13]. Recurrence occurred in our patient after three months and arthrodesis surgery was planned. When the literature is reviewed retrospectively, it can be seen that there are different arthrodesis techniques. Reconstruction with the biceps femoris muscle and the iliotibial band has been described. We performed tibiofibular arthrodesis using 3.5 cannulated screws and the fibular resection method described by Miskovski et al. [12], which allows joint motion and completely dries the source of the cyst.

## Conclusions

It should be kept in mind that synovial cysts developing on a chronic basis may cause progressive symptoms. The masses around the vessels, nerves, and tendons should be well evaluated and it should not be forgotten that these structures may cause symptoms and signs secondary to compression. In case of recurrence, complex options such as arthrodesis may be required. In addition, proximal tibiofibular joint arthrodesis is a valuable option in patients with recurrence. 
